# Does Vitamin D Concentration Matter? The Consequential Effects of Serum Vitamin D Concentration and Supplementation on Paediatric Fracture Risk

**DOI:** 10.3390/nu18040705

**Published:** 2026-02-22

**Authors:** Tan Si Heng Sharon, Eunice Anastasia Wilianto, Andrew Kean Seng Lim, James Hoipo Hui

**Affiliations:** 1 Division of Paediatric Orthopaedic Surgery, Department of Orthopaedic Surgery, National University Hospital, Singapore 119074, Singapore; andrew_ks_lim@nuhs.edu.sg (A.K.S.L.); jameshui@nus.edu.eg (J.H.H.); 2 Yong Loo Lin School of Medicine, National University of Singapore, Singapore 119228, Singapore; eunicewilianto@gmail.com

**Keywords:** paediatrics, orthopaedics, fractures, vitamin D, supplementation

## Abstract

Objective: The association between vitamin D status and paediatric fracture risk remains controversial, with inconsistent findings across existing studies. This study aimed to evaluate the relationship between serum 25(OH)D concentrations, vitamin D sufficiency, insufficiency and deficiency, vitamin D supplementation and fracture risk in a large Southeast Asian paediatric cohort. Methods: This retrospective cross-sectional study included children under 18 years whose serum 25(OH)D concentrations were measured between 2014 and 2022. One-way ANOVA determined statistical significance between 25(OH)D concentrations in fracture and non-fracture groups. Prevalence of vitamin D insufficiency, deficiency and supplementation was compared between the two groups. Chi-square tests evaluated the association between 25(OH)D concentrations and supplementation against fracture risk. Results: A total of 4530 children were included (157 fracture cases, 4373 controls). Mean serum 25(OH)D concentration was lower in the fracture group than in the controls (27.44 ± 12.26 vs. 30.75 ± 15.21 ng/mL; *p* = 0.007). Sub-sufficient vitamin D status (<30 ng/mL) was more prevalent among fracture patients (*p* = 0.001), and suboptimal (*p* = 0.001), insufficient (*p* = 0.001), and deficient (*p* = 0.014) categories were each significantly associated with fractures. An association between vitamin D supplementation and fracture risk was observed. However, the dataset did not permit the determination of causality and a protective effect cannot be inferred. Conclusions: Higher serum 25(OH)D concentrations were associated with lower fracture risk, suggesting that optimisation of vitamin D status may represent a modifiable factor in paediatric bone health. Healthcare institutions should aim to maintain adequate 25(OH)D concentrations (>30 ng/mL). An association between vitamin D supplementation and fracture risk was observed; however, causality cannot be inferred from this retrospective dataset.

## 1. Introduction

Fractures are common among the paediatric population, with a third of adolescents sustaining at least one before 17 years of age [1], due to porous bones and weak, growing unfused physes. Suffering a fracture comes with great inconvenience and disruption to their lives and, in unfortunate cases, brings long-term complications into adulthood like malunion, causing deformities, premature physeal closure and neurovascular damage.

Whether serum 25(OH)D concentrations protect against paediatric fractures is a highly discussed topic with conflicting views. T. Ergün and M. Cansever associate vitamin deficiency (≤20 ng/mL) with increased paediatric fracture risk, particularly in upper extremity fractures [2]. Other papers disagree with this view. N. Ramirez et al. reported no association between low 25(OH)D concentrations and fracture risk among Hispanic children [3]. Others suggest different ways in which vitamin D is related to fracture risk. B. Minkowitz et al. suggest vitamin D influences fracture severity rather than risk, where 25(OH)D <40 ng/mL increases relative risk of more severe fractures [4].

Vitamin D is an essential hormone in the body that is involved in crucial biological and metabolic processes for growth, skeletal development and survival. Vitamin D promotes intestinal absorption of calcium and phosphorus, maintaining serum mineral concentrations required for hydroxyapatite deposition within the bone matrix essential for normal bone mineralisation, growth and remodelling by osteoblasts and osteoclasts. In states of vitamin D insufficiency, reduced calcium absorption leads to secondary hyperparathyroidism, increased bone turnover, and preferential mobilisation of calcium from cortical bone, resulting in reduced bone mineral density and compromised bone strength. Insufficient vitamin D in the paediatric population may impair bone mineral accrual and compromise skeletal microarchitecture, resulting in brittle and weak bones that are more susceptible to fractures even in the absence of overt metabolic bone disease. In severe deficiency states, this can manifest in bone pathologies such as rickets, adolescent idiopathic scoliosis, spondylolysis and fractures [5,6,7]. Recent research in the adult population also discovered the role of vitamin D in glucose regulation, the immune system and disease prevention, reinforcing the extensive role of vitamin D in the body for maintenance and growth [8,9].

This study referenced guidelines used by the *Journal of Clinical Endocrinology & Metabolism* (JCEM) (Table 1).

Vitamin D is obtained through diet, sunlight exposure or supplements [9]. However, uncontrollable factors affecting vitamin D absorption, such as climate [10], culture (e.g., clothing preferences, diet) [11] and unforeseen circumstances (e.g., pandemic lockdowns) [12], hinder adolescents from obtaining sufficient vitamin D to build strong bones. In northern European countries and those with similar climates, vitamin D deficiency is very common, more so during winter and spring, due to the lack of year-round sunlight exposure [10,13,14]. In countries that generally dress more conservatively due to religion or cultural preferences, such as those in the Middle East, vitamin D deficiency is very prevalent despite their sunny climate, as coverings hinder adequate sunlight exposure [15], especially among girls [11].

A controllable way to increase 25(OH)D concentrations is by supplementation. The available guidelines on vitamin D supplementation mostly concern adults [16]. There are limited studies on what concentrations of vitamin D supplementation are effective and safe for the paediatric population. K. Middelkoop et al. found that 10,000 IU/week vitamin D supplementation for 3 years increased vitamin D status in HIV uninfected Black South African school children with low prevalence of vitamin D deficiency at baseline. However, effects did not impact bone mineral concentration, serum concentration of bone turnover markers or fracture risk [17]. Conversely, D. Ceroni et al. found that vitamin D supplements reduced seasonal fluctuations and are significantly associated with decreasing fracture risk [14]. Among the published literature, inconsistencies arise in their suggested amount, duration and context. This study aims to evaluate the association of 25(OH)D serum concentrations with fracture risk in paediatric patients and whether vitamin D supplementation can aid in lowering that risk. Interpretation of these divergent results is further limited by small sample sizes, which reduces statistical power, increasing susceptibility to random error and may inflate effect estimates, especially when deficiency prevalence is imbalanced between groups. Collectively, the existing literature points to support vitamin D as a context-dependent contributory factor rather than a consistent or independent determinant of paediatric fracture risk.

### 1.1. What Is Known

There are currently 23 studies on serum 25(OH)D and fractures in the available literature, with their sample size ranging from 46 to 1256. The existing literature demonstrates polarising findings regarding the association between vitamin D status and paediatric fracture risk. Several studies report cohorts with moderate to severe hypovitaminosis D having stronger associations. Others observe no significant differences between fracture and non-fracture groups, particularly those conducted in regions with high background prevalence of insufficiency.

### 1.2. What This Study Adds

This study, with 4530 participants from diverse Southeast Asian (SEA) backgrounds, is the largest cohort examining vitamin D and paediatric fractures. This is the first paper studying a SEA population. Research on vitamin D supplementation provides healthcare providers with clear evidence-based guidelines to boost the population’s serum 25(OH)D to prevent deficiency complications and improve supplementation recommendations.

## 2. Materials and Methods

This is a retrospective cross-sectional study of prospectively collected data of ambulatory children in a tertiary hospital in Singapore. A retrospective review was done on data collected from the hospital’s electronic database, which includes all paediatric patients below 18 years old who have serum 25(OH)D concentrations measured in the hospital between 2014 and 2022. Ethical approval for this retrospective study was granted by the National Healthcare Group Domain Specific Review Board. The requirement for informed consent was waived due to the retrospective nature of the study.

The data collected from each patient included age, history of diagnosed fracture, predisposing conditions to fracture, and whether vitamin D supplementation (inpatient and outpatient) was prescribed. Their baseline 25(OH)D concentration with date tested (patient’s first ever 25(OH)D measurement) and the subsequent 25(OH)D concentrations measured post supplementation with date tested were also collected. Serum 25(OH)D was measured in an accredited hospital laboratory using a standardised automated immunoassay with internal and external quality assurance. Patients with medical conditions that predispose the patient to a greater risk of fracture were excluded. The remaining paediatric patients were grouped into a case group with a history of fracture and a control group without a history of fracture. 25(OH)D concentrations and whether there was vitamin D supplementation were compared between the two groups.

The age of participants ranged from infancy to mid-adolescence. The majority experienced their fracture at a very young age. Early childhood was the most prevalent age range, with a significant cluster of fractures occurring between 2 and 8 years old. The youngest was approximately a month old, while the oldest participant was 15.16 years old. This pattern suggests the dataset particularly focuses on early childhood injuries.

Fractures analysed in this study reflect typical paediatric fractures presenting to a tertiary hospital setting. The fracture cohort represents a real-world paediatric population with fractures commonly encountered in routine clinical practice. Most paediatric fractures arise from trauma during normal activities (although in children, usually higher energy injuries than adults due to their activities) rather than underlying metabolic bone disease, fragility fractures and high energy accidents such as road traffic accidents. The type of fractures amongst participants was dominated by injuries to the long bones of the lower and upper limbs. Lower limb fractures were the most prevalent, with a very high volume of tibia/fibula, femoral, and ankle injuries, alongside numerous foot and toe fractures. Upper limb fractures were similarly prevalent, characterised by a high incidence of forearm (especially distal radius) and humeral breaks, with a notable cluster of paediatric style supracondylar humerus fractures, as well as many hand and finger fractures. Axial skeleton fractures involving the vertebral column, skull, and pelvis appear much less commonly. Beyond location, the composition was exclusively composed of closed fractures, though a few open fractures were noted.

Patients with medical conditions known to predispose to pathological fractures were excluded, including congenital skeletal disorders (e.g., osteogenesis imperfecta), metabolic bone disease (e.g., rickets), neuromuscular disorders, chronic renal or hepatic disease, malabsorption syndromes, endocrine disorders affecting bone metabolism, malignancy, and prolonged corticosteroid use. 

The patient supplementation data reveals that vitamin D supplementation was mostly in tablet or capsule form, while some were cholecalciferol in liquid oral form. The dosing was highly concentrated at 1000 IU, which was taken by the vast majority of those receiving supplementation. Beyond this standard dose, a smaller but notable number of patients were prescribed higher strengths, including 2000 IU and high-potency bolus doses such as 50,000 IU and 150,000 IU. These higher doses were possibly therapeutic treatment strategies for correction of severe insufficiency or deficiency or rapid repletion protocols, rather than routine supplementation. Other recorded dosages were infrequent, ranging from low doses like 400 IU to very high single doses like 300,000 IU. One entry for “6 mL” was noted but excluded from the dosage count as it represents a volume without a specified concentration. The vitamin D supplementation duration amongst participants reveals a highly polarised pattern. The vast majority of patients were supplemented for exactly 24 weeks, indicating this was likely a standard, pre-defined treatment or follow-up period in the institute. Outside of this primary cohort, durations were widely dispersed, ranging from very short periods of a few days to exceptionally long courses extending beyond a year, with the maximum recorded duration being approximately 396 weeks. The 1000 IU/24 weeks regimen appears to be the most common prescription of vitamin D supplementation.

### Statistical Analysis

Data was processed using SPSS Version 23. The mean, standard deviation, minimum and maximum of age distribution in the fracture cohort and serum 25(OH)D concentrations in fracture and non-fracture groups were calculated and compared. One-way ANOVA determined whether there was a statistically significant difference between serum 25(OH)D concentrations in different age groups and serum 25(OH)D concentrations in both fracture and non-fracture groups. Prevalence of vitamin D insufficiency and deficiency and vitamin D supplementation were compared. Chi-square tests were done to determine whether there was a significant association between serum 25(OH)D concentrations and different age groups, and vitamin D insufficiency and deficiency, and vitamin D supplementation and fracture risk. Logistic regression analysis was also done to determine the impact of 25(OH)D concentrations, vitamin D insufficiency, deficiency and vitamin D supplementation on the likelihood of a fracture and to quantify the relationship.

## 3. Results

### 3.1. Age Distribution and Stratification Within the Fracture Cohort

**Figure 1:**
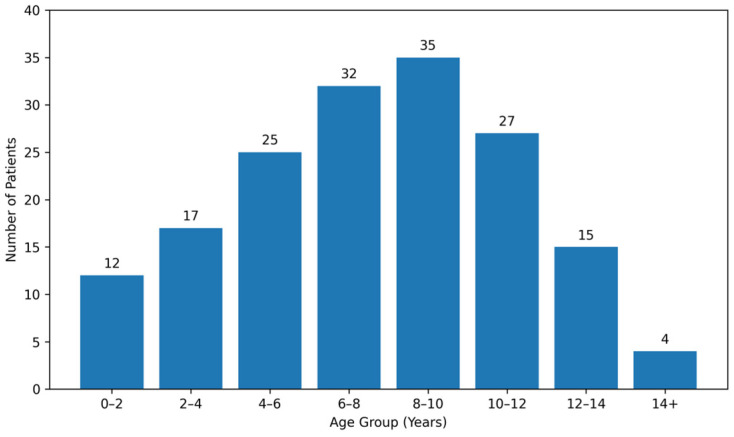
Age distribution histogram of fracture cohort.

Table 2 summarises the age distribution within the fracture cohort. Among the 157 children with fractures, the mean age was 7.87 ± 4.21 years (range: 0.01–15.16 years). Age stratification revealed that 69.4% (*n* = 109) of fractures occurred in school-age children (5–12 years), with peak incidence between 8 and 10 years (22.3%) as depicted in Figure 1. Younger children (<5 years) accounted for 18.5% (*n* = 29) of fractures, while adolescents (13–18 years) represented 12.1% (*n* = 19).

Age-stratified analysis of serum 25(OH)D concentrations among the 157 fracture patients revealed no significant differences between age groups. Mean vitamin D was 28.39 ± 12.54 ng/mL in children <5 years (*n* = 29), 27.06 ± 12.15 ng/mL in those aged 5–12 years (*n* = 109), and 27.18 ± 12.92 ng/mL in adolescents aged 13–18 years (*n* = 19). One-way ANOVA confirmed no statistically significant differences (F = 0.18, *df* = 2154, *p* = 0.834). Vitamin D deficiency (<20 ng/mL) was present in 24.1%, 26.6%, and 31.6% of each age group, respectively. The chi-square test shows no significant difference in status distribution (χ^2^ = 0.76, *p* = 0.944) (Table 3).

### 3.2. The Serum 25(OH)D Concentrations in Fracture and Non-Fracture Groups

**Figure 2:**
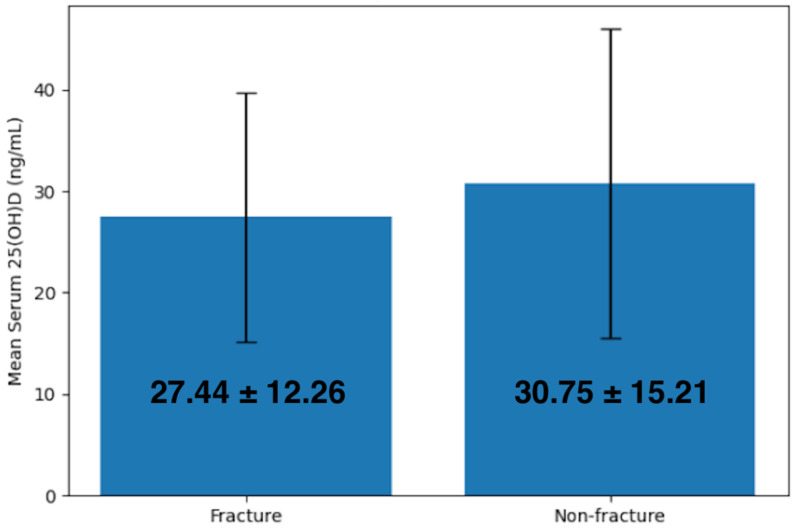
Graphical representation of serum 25(OH)D concentrations in fracture and non-fracture groups.

As shown in Table 4, the mean 25(OH)D concentration among all the participants included was 30.64 ng/mL ± 15.13. 25(OH)D concentration in the fracture group (27.44 ng/mL ± 12.26) was lower than that in the non-fracture group (30.75 ng/mL ± 15.21), and the difference was statistically significant (*p* = 0.007) (Figure 2). A logistic regression analysis demonstrated odds of fracture decreasing by 1.7% for each unit increase in serum 25(OH)D and the results were statistically significant (B = 0.017, OR = 1.017, 95% CI = 1.005 to 1.030, *p* = 0.007).

### 3.3. Prevalence of Vitamin D Insufficiency (25(OH)D Concentrations 20–30 ng/mL) and Deficiency (25(OH)D Concentrations < 20 ng/mL) in Fracture and Non-Fracture Groups

**Figure 3:**
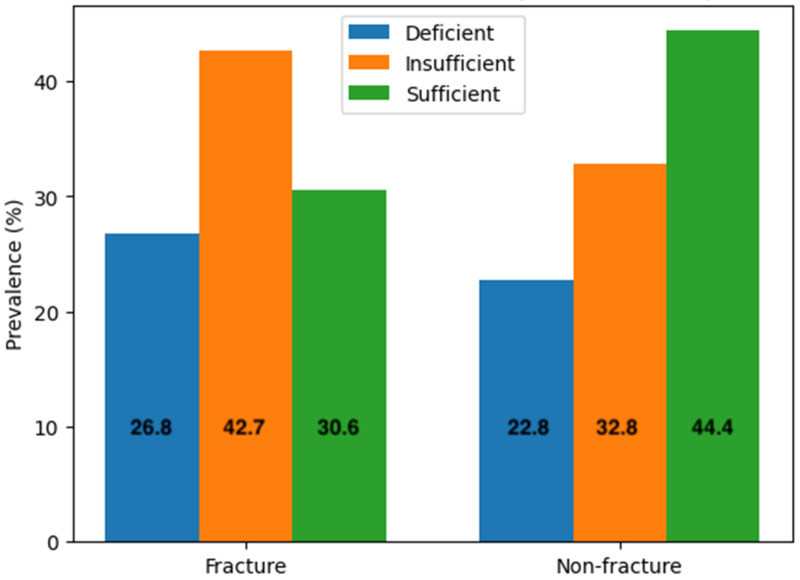
Graphical representation of the distribution of participants with different vitamin D status in fracture and non-fracture groups.

As shown in Table 5 and Figure 3, among the 157 participants in the fracture group, 42 (26.7%) had deficient 25(OH)D concentrations of equal or less than 20 ng/mL, 67 (42.7%) had insufficient 25(OH)D concentrations between 20 and 30 ng/mL and 48 (30.6%) had sufficient 25(OH)D concentrations of equal or more than 30 ng/mL. In the non-fracture group of 4373 participants, 999 (22.8%) were deficient, 1434 (32.8%) had insufficient 25(OH)D concentrations and 1940 (44.4%) had sufficient 25(OH)D concentrations. There was a greater percentage of participants among the fracture group with 25(OH)D concentrations below sufficient concentrations compared to the non-fracture group (*p* = 0.001) (Table 6). A logistic regression analysis demonstrated statistically significant results where odds of having a fracture decrease by approximately 44.7% when serum 25(OH)D concentrations are normalised from a less than sufficient concentration (B = −0.594, OR = 0.553, 95% CI = 0.391 to 0.780, *p* = 0.001).

As shown in Table 7, the fracture group had a greater and significant proportion of vitamin D-deficient individuals compared to the non-fracture group (*p* = 0.013). Logistic regression analysis demonstrated odds of suffering a fracture decreasing significantly by approximately 41.2% if serum 25(OH)D concentrations reach the normal range from a deficient range (B = −0.531, OR = 0.588, 95% CI = 0.386 to 0.893, *p* = 0.014).

As shown in Table 8, there is a significantly greater number of participants who had insufficient 25(OH)D concentrations in the fracture group (*p* = 0.001). Logistic regression analysis demonstrated odds of suffering a fracture decreasing significantly by approximately 47.1% if serum 25(OH)D concentrations reach the normal range from an insufficient range (B = −0.636, OR = 0.529, 95% CI = 0.391 to 0.781, *p* = 0.001).

### 3.4. Vitamin D Supplementation Among Participants in Fracture and Non-Fracture Groups

**Figure 4:**
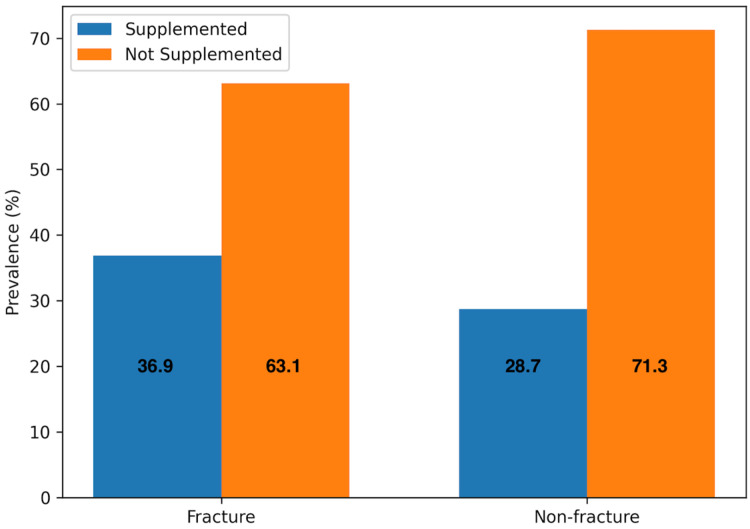
Graphical representation of vitamin D supplementation in fracture and non-fracture groups.

From Table 9, the fracture group had 58 participants who had vitamin D supplementation (36.9%) and 99 participants without (63.1%). In the non-fracture group, 1253 had vitamin D supplementation (28.7%), and 3120 did not receive vitamin supplementation from the hospital (71.3%) (Figure 4).

Conversely, out of all the 1311 participants who received vitamin D supplementation, 4.4% had sustained a fracture, while 95.6% of them did not. 3219 participants did not receive vitamin D supplementation and of them, the fracture and non-fracture groups made up 3.1% and 96.9%, respectively.

The Pearson chi-square test revealed a chi-square value of 5.064 and *p* = 0.026, indicating a statistically significant association between vitamin D supplementation and fractures. An association between vitamin D supplementation and fracture risk was observed. However, the dataset did not permit the determination of causality or temporal direction. It therefore cannot be concluded whether supplementation was a cause or consequence of fracture, and a protective effect cannot be inferred.

## 4. Discussion

This retrospective study aims to firstly study the 25(OH)D concentrations of the participants in the fracture and non-fracture groups to find an association with fracture risk. Secondly, a comparison was conducted of those who were prescribed vitamin D supplements and those without, to find an association between supplementation and paediatric fracture risk. The results show that lower 25(OH)D concentrations increase paediatric fracture risk and 25(OH)D supplementation reduces the risk of paediatric fractures.

### 4.1. Age Distribution Within the Fracture Cohort

Analysis revealed no significant age-related differences in serum 25(OH)D concentrations among fracture patients. These findings challenge the assumption that vitamin D status follows predictable age-related patterns in children sustaining fractures. Rather, they suggest that vitamin D insufficiency represents a pervasive, age-independent risk factor in this population. The lack of significant variation implies that targeted interventions should not focus exclusively on specific age brackets but rather adopt a universal approach to vitamin D optimisation across paediatric care. This was particularly relevant given that children 5–12 years old constituted 69.4% of our fracture cohort yet showed similar deficiency rates to younger and older groups. The homogeneity of 25(OH)D status across ages underscores its role as a modifiable risk factor independent of developmental stage.

### 4.2. 25(OH)D Concentration and Paediatric Fracture Risk

Findings from this paper support the claim that adequate 25(OH)D concentrations have protective effects against fractures. This was in line with other papers, such as the one published in 2016 by N. M. Al-Daghri et al. [18], which reported serum 25(OH)D being clearly lower among participants with a fracture history than those without. It added that age and gender are factors that affect 25(OH)D concentrations and paediatric fracture risk, where the association between age and 25(OH)D concentrations was positive in boys and negative in girls. There was a higher prevalence of fractures in boys than in girls.

However, there are papers whose results show no association between 25(OH)D concentrations and fracture risk. A J Karkenny et al. (2023) [19] reported that low 25(OH)D concentrations are common in children with fractures, but there was no difference in 25(OH)D concentrations between case and control groups. It writes that certain genotypes are associated with higher 25(OH)D concentrations, while others are associated with fracture risk independent of 25(OH)D concentrations, highlighting that there are many factors related to 25(OH)D concentrations and controlling confounders that would advance our understanding of the potential association [19]. Many studies have focused on populations in a certain area, where the country studied is mostly made up of a racially homogenous population, the genetic pool may be more skewed. In a paper by L N. Anderson et al., they found that serum 25(OH)D concentrations at the time of fracture may not be associated with fracture risk in the paediatric population with relatively high 25(OH)D concentrations. It also acknowledged that the study population is relevant, where the sample studied was skewed and made up mostly of participants with sufficiently high 25(OH)D concentrations [20].

A significant factor distinguishing this study from others is the large sample size, consisting of 4530 participants. This study benefits from greater statistical power, reliability and minimises the impact of confounding factors that could disproportionately affect outcomes in smaller sample groups. The discrepancies in findings may be attributable to the differential reliability associated with variations in sample size. Many of the studies reporting no association between 25(OH)D and fracture risk had sample sizes significantly smaller, averaging less than 500 participants. This potentially limits their capacity to identify nuanced but clinically relevant associations. Additionally, inconsistencies in 25(OH)D cut-off concentrations across these studies further complicate direct comparisons. This paper, having a significantly larger, diverse sample and internationally recognised standardised 25(OH)D thresholds, provides a comprehensive and reliable evaluation of 25(OH)D’s role in fracture risk among children.

### 4.3. 25(OH)D Insufficiency and Deficiency

Healthcare institutes should aim to achieve at least adequate 25(OH)D concentrations (>30 ng/mL) to make a significant difference in protective effects against fractures. Existing literature by B. Minkowitz et al. supports this and even advised minimal target 25(OH)D concentration for paediatrics to be ≥40 ng/mL, as the study found that concentrations between 30 and 40 ng/mL places children at an increased risk of severe fractures compared with children with 25(OH)D ≥ 40 ng/mL [4]. Endocrine Practice Guidelines also recommended 40 to 60 ng/mL as the target concentrations for serum 25(OH)D concentrations. Analysis from this paper reports a greater proportion of participants from the fracture group with deficient or insufficient 25(OH)D concentrations compared to the non-fracture group, and it is statistically significant (Table 6, Table 7 and Table 8). The use of an insufficiency cut off, namely >30 ng/mL, would be more appropriate as a suggested 25(OH)D concentration protective against paediatric fracture. Applying a deficiency threshold alone would attenuate the association and exclude a substantial proportion of participants with suboptimal vitamin D status. Given that insufficiency concentration would capture a larger at-risk population while maintaining statistical significance, it represents a more clinically meaningful threshold when evaluating paediatric fracture risk.

### 4.4. 25(OH)D Supplementation and Fracture Risk

There have been extensive studies on 25(OH)D supplementation in adults and how it affects bone density and fracture risk, but much less for the paediatric population. With the importance of 25(OH)D in maintaining healthy bone physiology and other roles it performs in the body, there is a need to research how best to increase serum 25(OH)D concentrations, especially for patients with 25(OH)D concentrations at higher risk of fractures.

There have been studies that report no association between vitamin D supplementation and fracture risk. This paper describes an association between vitamin D supplementation and lower fracture risk—contrast with recent large RCTs. Middelkoop (2024) and D. Ganmaa et al. report weekly oral administration of 10,000 IU and 14,000 IU vitamin D3, respectively, to participants with insufficient baseline serum 25(OH)D, for three years, was effective in increasing serum 25(OH)D concentration into the physiologic range, but does not decrease fracture risk or increase bone mineral density [17,21].

Current clinical guidelines for paediatric vitamin D supplementation primarily focus on preventing deficiency and rickets rather than fracture prevention. The American Academy of Paediatrics (AAP) recommends a daily intake of 400 IU for infants and 600 IU for children and adolescents to maintain bone health [22], while the Society for Adolescent Health and Medicine suggests 600 IU daily for healthy adolescents, and at least 1000 IU daily for adolescents who are at risk for vitamin D insufficiency [23]. The Global Consensus Recommendations on nutritional rickets emphasise that vitamin D supplementation should be combined with adequate calcium intake for optimal bone mineralisation [24]. Notably, these guidelines do not endorse high-dose or long-term vitamin D supplementation solely for fracture prevention in otherwise healthy children.

The baseline 25(OH)D concentration of the sample studied may affect the effectiveness of vitamin D supplementation in increasing 25(OH)D concentrations. It might be so that supplementation would only make a significant difference in children with a lower baseline of 25(OH)D, where dietary vitamin D supplements would be more readily absorbed and contribute to serum concentrations. In a review by R J. Moon et al., it suggests with borderline significance that vitamin D supplementation in children with the lowest 25(OH)D concentrations <14 ng/mL would benefit the whole body and lumbar spine BMD [25].

However, this paper reports an association between vitamin D supplementation and fractures. This is against the backdrop of our sample mean being borderline adequate at 30.64 ng/mL. This paper captures both routine supplementation and therapeutic repletion. Accordingly, there is an association between participants receiving vitamin D supplementation and fracture risk, but it may not be causative. The study did not analyse serum 25(OH)D concentrations pre- and post-supplementation stratified by supplementation status, had limited insight into prescribing indications, and the retrospective dataset lacked information on adherence to supplementation. These limitations introduce potential confounding, thus findings should be interpreted as associative rather than evidence of an independent protective effect.

This study differs from the aforementioned RCTs in design, population and baseline vitamin D status. Compared to other published studies from other countries, the sample has a higher mean 25(OH)D concentration compared to seasonal or less developed countries. The regional context may partly explain the findings observed in this study. The sample, made up of participants living in Singapore, a tropical country near the equator with generally sunny weather all year round, provides residents with adequate sun exposure to produce 25(OH)D. Despite residing in equatorial regions with abundant year-round sunlight, vitamin D insufficiency remains prevalent among children in Southeast Asia, including in Singapore. This paradox has been attributed to urban living, high indoor academic demands, reduced outdoor physical activity due to the heat or the availability of convenient public transport underground, widespread sunscreen use, and cultural clothing practices that limit effective ultraviolet exposure. Due to the competitive nature of an Asian country, available sunlight may not correlate directly to higher 25(OH)D concentrations, as competitive academics at a young schooling age may compromise outdoor activity time in children studying in Singapore. Additionally, dietary vitamin D intake in Southeast Asian populations is often suboptimal, as commonly consumed diets are not routinely fortified with vitamin D and may be relatively low in calcium as compared to their Western counterparts. However, Singapore, being a first-world country, also has the buying power to access supplements and vitamin D-rich diets as a means to gain vitamin D, leading to relatively better 25(OH)D concentrations. Singapore’s Ministry of Health and Ministry of Education also places emphasis on managing the BMI of school children in Singapore through extensive education on a healthy diet and exercise and helping overweight children lose weight by putting them on exercise programmes. These factors may contribute to marginal vitamin D status even in otherwise healthy children, potentially influencing skeletal health and fracture susceptibility.

### 4.5. Strengths and Limitations

This study has one of the largest sample sizes among available literature, allowing for sufficient statistical power to detect meaningful differences between the association of fracture risk and vitamin D and its supplementation.

There are limited studies done among Asian populations. This is the first paper researching vitamin D and its supplementation among the paediatric population and their fracture risks in SEA. This is especially relevant due to the common prevalence of vitamin D deficiency among healthy children living in the SEA region [26]. The sample studied consists of residents in Singapore, which is known to be diversely multiracial, a good representation of the under-reported SEA region that shares similar racial compositions and climate.

However, this study has limitations that merit discussion. This paper did not consider confounders that may have an effect on serum 25(OH)D concentrations, but focused on just vitamin D supplementation. However, there is existing literature that extensively covers the effect of gender, race, diet, sun exposure and other factors, some of which should also be considered. To ensure a more accurate association between supplementation and fracture risk, compliance with vitamin D supplements prescribed should be confirmed along with noting other vitamin D or other forms of supplementation taken outside of prescription.

A study exploring other supplements involved in bone metabolism, such as calcium and its supplementation on fracture risk, should also be explored to study whether synergistic supplementation can increase effectiveness. Key biochemical markers of bone metabolism, including serum calcium, phosphate, and parathyroid hormone, were not routinely available in this cohort and were therefore not analysed. Future prospective studies could consider incorporating comprehensive biochemical profiling. Future prospective studies could incorporate fracture classification to further refine the observed association, reporting fracture mechanism and anatomical site. Although lifestyle (e.g., BMI, sun exposure, physical activity, etc) and socioeconomic factors have been extensively examined in prior studies, these variables were not consistently available in this retrospective dataset and could be incorporated in future prospective studies of this scale to further refine the observed associations. Factors of supplementation regimen, such as duration, dosage, type, route of supplementation, and their effect on 25(OH)D concentrations and fracture risk, could also be studied. This could be tested on groups of different vitamin D status to see if supplementation has different effectiveness in increasing 25(OH)D concentrations. Given this paper’s observational study design, the observed association between vitamin D supplementation and fracture risk does not imply a causal relationship.

## 5. Conclusions

This literature produced findings supporting an association between serum 25(OH)D concentrations and paediatric fracture risk, with higher serum 25(OH)D concentrations, ideally at a sufficient range, having a protective effect against fracture risk. As evident in the results, a way to decrease fracture risk is by vitamin D supplementation, where an ideal sufficient serum 25(OH)D concentration has protective effects against fracture risk.

## Figures and Tables

**Table 1:** JCEM guidelines.

Vitamin D Status	Range of Serum 25(OH)D Concentration
Deficient	<20 ng/mL (<50 nmol/mL)
Insufficient	20 to <30 ng/mL (50 to <75 nmol/mL)
Sufficient	≥30 ng/mL (≥75 nmol/mL)

**Table 2:** Age distribution within the fracture cohort.

Age Group	N (%)	Mean Age ± SD	Mean Serum 25(OH)D Concentration ± SD	Range	Median
<5 years	29 (18.5)	2.89 ± 1.67	28.39 ± 12.54	0.01–4.84	3.03
5–12 years	109 (69.4)	8.48 ± 2.19	27.06 ± 12.15	5.00–12.99	8.49
13–18 years	19 (12.1)	14.02 ± 1.02	27.18 ± 12.92	13.00–15.16	14.21
Total	157 (100)	7.87 ± 4.21	27.44 ± 12.26	0.01–15.16	8.02

**Table 3:** Distribution of Vitamin D status prevalence by age.

Age Group	Deficient (<20 ng/mL) (%)	Insufficient (20–30 ng/mL) (%)	Sufficient (≥30 ng/mL) (%)
<5 years	7/29 (24.1)	11/29 (37.9)	11/29 (37.9)
5–12 years	29/109 (26.6)	44/109 (40.4)	36/109 (33.0)
13–18 years	6/19 (31.6)	6/19 (31.6)	7/19 (36.8)
Total	42/157 (26.8)	61/157 (38.9)	54/157 (34.4)

**Table 4:** Mean serum 25(OH)D concentrations in fracture and non-fracture groups.

	Fracture Group (N = 157)	Non-Fracture Group (N = 4373)	Overall	One-Way ANOVA, *p*-Value	Odds Ratio (95% CI for Exp(B))
25(OH)D Concentration, ng/mL Mean (SD)	27.44 (12.26)	30.75 (15.21)	30.64 (15.13)	0. 007	1.017 (1.005–1.03) *p* = 0.007

**Table 5:** Distribution of participants of different vitamin D status in fracture and non-fracture groups.

	Fracture Group (N = 157)	Non-Fracture Group (N = 4373)	Overall
Number of participants with deficient vitamin D [≤20 ng/mL] (%)	42 (26.7)	999 (22.8)	1041
Number of participants with insufficient vitamin D [20–30 ng/mL] (%)	67 (42.7)	1434 (32.8)	1501
Number of participants with sufficient vitamin D [≥30 ng/mL] (%)	48 (30.6)	1940 (44.4)	1988
Total	157 (100)	4373 (100)	4530

**Table 6:** Vitamin D deficiency and insufficiency in fracture and non-fracture groups.

	Fracture Group (N = 157)	Non-Fracture Group (N = 4373)	Overall	Chi-Square Tests, *p*-Value	Odds Ratio (95% CI for Exp(B))
Number of participants with deficient + insufficient vitamin D [<30 ng/mL] (%)	109 (69.4)	2433 (55.6)	2542	0.001	1.81 (1.28–2.56) *p* = 0.001
Number of participants with sufficient vitamin D [≥30 ng/mL] (%)	48 (30.6)	1940 (44.4)	1988		

**Table 7:** Vitamin D deficiency in fracture and non-fracture groups.

	Fracture Group (N = 157)	Non-Fracture Group (N = 4373)	Overall	Chi-Square Tests, *p*-Value	Odds Ratio (95% CI for Exp(B))
Number of participants with deficient vitamin D [≤20 ng/mL] (%)	42 (26.8)	999 (22.8)	1041	0.014	1.70 (1.12–2.59) *p* = 0.013
Number of participants with sufficient vitamin D [≥30 ng/mL] (%)	48 (30.6)	1940 (44.4)	1988		

**Table 8:** Vitamin D insufficiency in fracture and non-fracture groups.

	Fracture Group (N = 157)	Non-Fracture Group (N = 4373)	Overall	Chi-Square Tests, *p*-Value	Odds Ratio (95% CI for Exp(B))
Number of participants with insufficient vitamin D [20–30 ng/mL] (%)	67 (42.7)	1434 (32.8)	1501	0.001	1.89 (1.39–2.75) *p* = 0.001
Number of participants with sufficient vitamin D [≥30 ng/mL] (%)	48 (30.6)	1940 (44.4)	1988		

**Table 9:** Vitamin D supplementation in fracture and non-fracture groups.

	Fracture Group (N = 157)	Non-Fracture Group (N = 4373)	Overall	Chi-Square Tests, *p*-Value	Odds Ratio (95% CI for Exp(B))
Number of participants with Vitamin D supplementation (%)	58 (36.9)	1253 (28.7)	1311	0.026	0.685 (0.492–0.954) *p* = 0.025
Number of participants without Vitamin D supplementation (%)	99 (63.1)	3120 (71.3)	3219		

## Data Availability

The raw data supporting the conclusions of this article will be made available by the authors on request.
